# Evolutionary dynamics of host specialization in wood-decay fungi

**DOI:** 10.1186/s12862-018-1229-7

**Published:** 2018-08-03

**Authors:** Franz-Sebastian Krah, Claus Bässler, Christoph Heibl, John Soghigian, Hanno Schaefer, David S. Hibbett

**Affiliations:** 1 Plant Biodiversity Research Group, Center for Food and Life Sciences Weihenstephan, Technische Universität München, Freising, Germany; 2 Baverian Forest National Park, Grafenau, Germany; 3 Department of Environmental Science, The Connecticut Agricultural Experiment Station, New Haven, CT 06511 USA; 4 Biology Department, Clark University, Worcester, MA 01610 USA

**Keywords:** Wood-decay fungi, Decay mode, White rot, Brown rot, Host specialization, R package rusda

## Abstract

**Background:**

The majority of wood decomposing fungi are mushroom-forming Agaricomycetes, which exhibit two main modes of plant cell wall decomposition: white rot, in which all plant cell wall components are degraded, including lignin, and brown rot, in which lignin is modified but not appreciably removed. Previous studies suggested that brown rot fungi tend to be specialists of gymnosperm hosts and that brown rot promotes gymnosperm specialization. However, these hypotheses were based on analyses of limited datasets of Agaricomycetes. Overcoming this limitation, we used a phylogeny with 1157 species integrating available sequences, assembled decay mode characters from the literature, and coded host specialization using the newly developed R package, *rusda*.

**Results:**

We found that most brown rot fungi are generalists or gymnosperm specialists, whereas most white rot fungi are angiosperm specialists. A six-state model of the evolution of host specialization revealed high transition rates between generalism and specialization in both decay modes. However, while white rot lineages switched most frequently to angiosperm specialists, brown rot lineages switched most frequently to generalism. A time-calibrated phylogeny revealed that Agaricomycetes is older than the flowering plants but many of the large clades originated after the diversification of the angiosperms in the Cretaceous.

**Conclusions:**

Our results challenge the current view that brown rot fungi are primarily gymnosperm specialists and reveal intensive white rot specialization to angiosperm hosts. We thus suggest that brown rot associated convergent loss of lignocellulose degrading enzymes was correlated with host generalism, rather than gymnosperm specialism. A likelihood model of host specialization evolution together with a time-calibrated phylogeny further suggests that the rise of the angiosperms opened a new mega-niche for wood-decay fungi, which was exploited particularly well by white rot lineages.

**Electronic supplementary material:**

The online version of this article (10.1186/s12862-018-1229-7) contains supplementary material, which is available to authorized users.

## Background

About 2000 billion tons of carbon is present in terrestrial ecosystems [1], of which 550 billion tons are fixed in vegetation [2]. In forest ecosystems, most plant biomass is stored in the form of dead wood [3]. Woody plant cell walls consist mainly of the lignocellulose complex which is composed of the polymeric polysaccharides cellulose, hemicellulose and lignin heteropolymers [4, 5]. Cellulose is a macropolymer consisting of linear chains of glucose subunits that can take on a recalcitrant crystalline form [6]. Hemicelluloses are matrix polysaccharides consisting of various heteropolymers, e.g., of xylans and glucomannans [7]. Lignin is a complex aromatic polymer that is resistant to hydrolytic degradation [8]. The amount of cellulose in woody plants is 40–50% of the wood dry weight and for hemicelluloses and lignin 15–30% each. The plant biomass further consists of macromolecules such as lipids, waxes, proteins and phenolic compounds [3]. The most efficient agents of the decay of the lignocellulose complex are saprotrophic fungi, which therefore play pivotal roles in the cycling of carbon [9] and nutrients [10] in the forest ecosystem. Wood is produced by angiosperms and gymnosperms, which together comprise more than 60,000 species [11]. Angiosperms regularly have lower amounts of lignin than gymnosperms, whereas angiosperms regularly have higher amounts of cellulose than gymnosperms [12, 13]. Further, angiosperms often have lower amounts of non-structural secondary compounds (plant extractives) than gymnosperms [12–15], with some exceptions, e.g., species of the genera *Quercus*, *Fagus* or *Malus* [16].

**Fig. 1:**
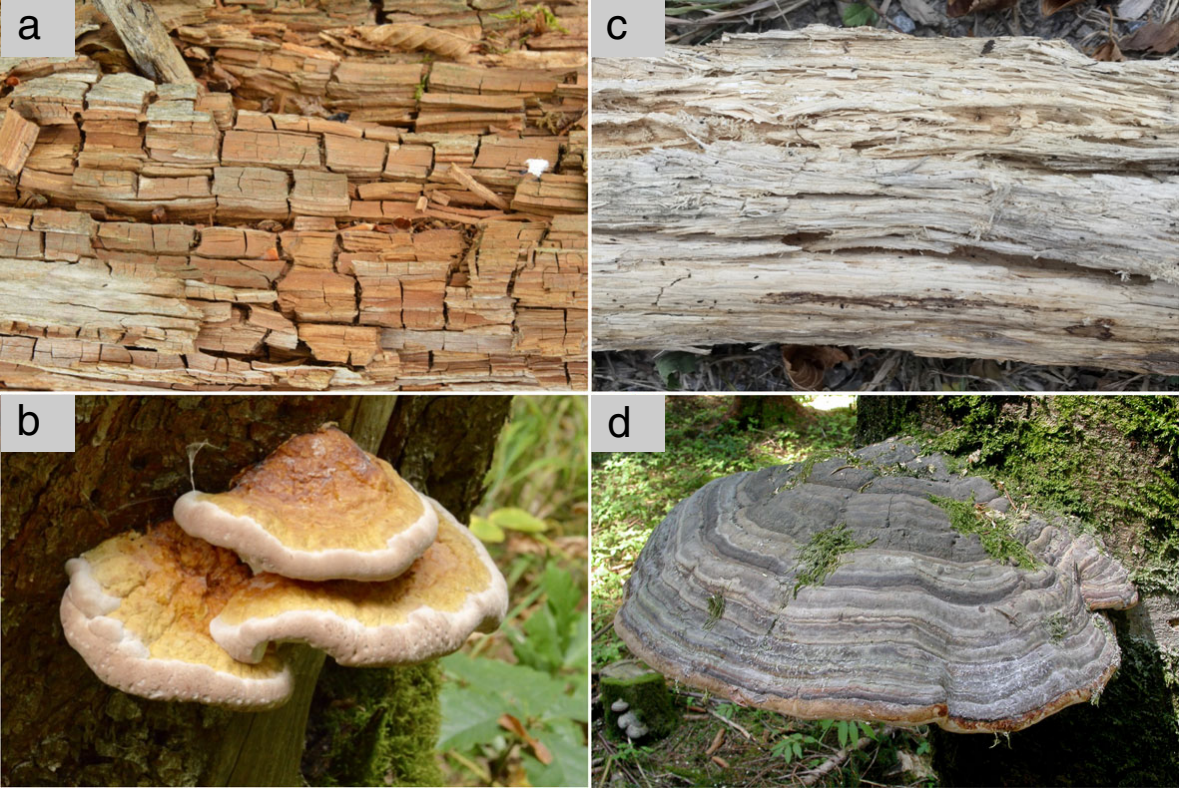
Brown and white rot residues and fungal fruit bodies. **a**) Brown rot residue, **b**) brown rot fungus, *Fomitopsis pinicola* (Polyporales, Fomitopsidaceae), **c**) white rot residue, **d**) white rot fungus, *Fomes fomentarius* (Polyporales, Polyporaceae)*.* Photos by F.-S. Krah (**a**,**b**,**c**) and Heinrich Holzer (**d**)

The main agents of wood decay are members of the class Agaricomycetes (Basidiomycota). Agaricomycetes contains about 21,000 species with a worldwide distribution, including many lifestyles, e.g. mycorrhizal symbionts, pathogens, and saprotrophs. Most saprotroph fungi within the Agaricomycetes are dead wood decaying fungi. Dead wood decay modes can be classified as either white or brown rot. Brown rot fungi attack cellulose but do not significantly degrade lignin [17], resulting in a brownish residue that breaks into cubical fragments, whereas white rot fungi degrade both cellulose and lignin [18], leaving a bleached fibrous residue (Fig. 1). Hemicellulose can be degraded by both brown and white rot fungi [19]. Whereas dead wood is mainly decayed by Agaricomycetes, in plant litter decay, Ascomycota play a significant role along with litter-decomposing Agaricomycetes [19, 20]. Other decay modes are also present, such as “soft rot” in some Ascomycota and “grey rot” in some Basidiomycota such as *Schizophyllum commune* [21]. Although many ectomycorrhizal fungi are partially saprotrophic, their decay abilities are considered marginal compared to wood decay fungi [22, 23].

The enzymatic basis of the differences between white rot and brown rot has been studied extensively in comparative genomic analyses [21, 24–26]. White rot fungi are distinguished by high copy numbers of genes encoding different carbohydrate-active enzymes (CAZymes) which are classified based on the CAZy database [27]. In general, CAZymes, which act on crystalline cellulose are more abundant in white rot genomes compared with brown rot [24]. Glycoside hydrolase (GH) families (e.g., GH6 and GH7, including cellobiohydrolases) are more abundant in white rot compared with brown rot fungi [24]. Further, lytic polysaccharide monooxygenases (LPMOs) from the AA9 family are more abundant in white than brown rot fungi [24]. Apart from Agaricomycetes, LPMOs can be found in Ascomycetes and Mucoromycotina [21, 24]. Finally, lignin-degrading class II peroxidases (AA2) and other heme-containing peroxidases are more common in white rot, and reduced or absent in brown rot fungi [26] (for mechanisms of action see Hofrichter et al. [28]). The most recent common ancestor of Agaricomycetes was a white rot species (based on an inferred expansions of AA2 and other lignocellulolytic enzymes) with at least four independent origins of brown rot, correlated with parallel losses of genes encoding diverse CAZys, and the complete loss of ligninolytic class II peroxidases (AA2) [24], making reversals to white rot unlikely. This white rot ancestor likely lived roughly 290 (+/− ca. 70) million years ago (MYA) [24]. Analyses of a sample of 62 genomes by Nagy et al. [29] suggested that expansions of cellobiohydrolases (GH6, GH7), LPMOs (AA9), and other plant cell wall degrading enzymes occurred early in the evolution of Agaricomycetes, prior to the expansion of class II peroxidases (AA2).

Gilbertson [30] investigated ecological differences between white and brown rot decay modes, noting that brown rot fungi preferentially occur on gymnosperm hosts [31]. Gilbertson thus suggested a correlated evolution of brown rot decay mode and gymnosperm specialization. Hibbett and Donoghue [32] tested Gilbertson’s hypothesis using phylogenetic comparative methods. Their results suggested that the evolution of brown rot was correlated with the evolution of exclusive decay of gymnosperm hosts. However, this inference was made from a dataset with limited taxonomic sampling, with only a total of 130 species [32].

To assess the evolution of decay modes and patterns of host specialization among wood decay fungi in Agaricomycetes, we utilized a time-calibrated mega-phylogeny approach and drew on the extensive Fungus-Host Distribution Database built by the United States Department of Agriculture (USDA) [33]. We then used this mega-phylogeny and host associations, which encompassed 1157 species from 14 orders, to test two hypotheses: (1) brown rot fungi occur primarily on gymnosperm hosts; and (2) brown rot fungi switched more frequently towards gymnosperm hosts than white rot lineages. We further use this large-scale dataset to investigate white rot specialization pattern and mechanisms, a topic currently neglected due to a focus on specialization pattern of brown rot fungi.

## Methods

### Trait data and character matrix

To test our hypotheses, we gathered data on decay mode and host associations for Agaricomycetes. For decay mode, we used the “decay.type” as published in Tedersoo et al. [34], which is available on the genus level, and we also conducted a literature search for additional genera. Tedersoo et al. (2014) investigated lifestyle-dependent global fungal diversity and therefore coded the trophic status (six states, e.g., biotroph), the lifestyle (17 states, e.g., ectomycorrhizal) and decay type (four states, e.g. white rot, brown rot) for more than 10,000 genera. We used only species with either white or brown rot in our analysis and excluded other lifestyles (e.g., mycorrhizal). This gave us the decay mode of particular species in the genera. We then extrapolated this decay mode to the remaining species of a genus (with one exception, see below). Our justification is that decay mode has often been a focus of taxonomists and thus was widely used to distinguish genera such as *Antrodia* and *Antrodiella* [35]*, Lentinus* and *Neolentinus-Heliocybe* [36], and *Daedalea* and *Daedaleopsis* [37]. We found only three genera where more than one decay mode has been reported: *Clitocybula* [38, 39], *Hyphoderma* [40]*,* and *Mucronella* [40, 41]. *Clitocybula* and *Mucronella* were deleted from the dataset because no host data were available. For *Hyphoderma* we used only the two species where decay mode references were found. To estimate how this strategy might affect our interpretations, we re-sampled a single species per genus from our final dataset (hereafter “one-genus-subset”) and repeated the analyses described below 100 times.

To gather data on host associations, we used the R package “rusda”, written for this study, as an interface to the USDA Fungus-Host Distribution Database (FHDD) [33]. The FHDD contains fungus-host combinations, but does not provide information on the occurrence frequencies on a particular host (other than the number of published records on each host). The “rusda” package makes it possible to retrieve (“query”) host data for fungal species, and vice versa. For a detailed description, basic usage and evaluation of the R package “rusda”, see Additional file 1 and for repositories refer to Availability of data and material.

To retrieve host associations from the FHDD we used the function *associations*, which takes an input of species names and provides an output list of fungus-host combinations. As input we used the NCBI taxonomy for fungi and re-classified the order level where necessary (Additional file 1: Table S3). We then produced a dataset of plant phyla by matching host genera to the Spermatophyta taxonomy downloaded from NCBI taxonomy using the R package “megaptera” [42]. Thus, we retrieved the phylum information “Acrogymnosperma” and “Magnoliophyta” for each host species. We refer to “Magnoliophyta” as angiosperm (A) and “Acrogymnosperma” as gymnosperm (G) and stored the number of gymnosperm and angiosperm associations for each fungus species in a table. Species which did not belong to either Acrogymnosperma or Magnoliophyta were deleted from the dataset. We further deleted all non-woody plants based on the woodiness dataset which classified more than 35,000 plants into woody and non-woody [43]. Thus, the final host dataset included only woody plants from Acrogymnosperma or Magnoliophyta; seedless vascular plants, bryophytes, algae, and non-plant hosts were excluded. The FHDD covers mainly temperate North America and Europe [33].

The host association data were used to calculate the number of angiosperm and gymnosperm host species for each fungus species. We defined the “gymnosperm association” by dividing the number of gymnosperm host tree species (N_G_) by the sum of the number of angiosperm (N_A_) and gymnosperm host tree species: gymnosperm associations [%] = N_G_/(N_G_ + N_A_). Thus, a gymnosperm association of 100% means that a fungus is reported exclusively on gymnosperm hosts in the Fungus-Host database, whereas 0% means only angiosperm hosts are reported. We classified host preferences into three states: (1) generalism, (2) angiosperm specialization, or (3) gymnosperm specialization. Based on the distribution of gymnosperm association [%] (Fig. 4c), we defined specialization based on the gymnosperm association [%] with a threshold of ≥90% for gymnosperm specialization and a threshold of ≤10% for angiosperm specialization (hereafter “90–10 specialization”). Previous studies used exclusivity as a measure of host association [32], but missing or incorrect data for a single fungus observation may then lead to misclassification of a species. Nonetheless, we also inferred our final model (see Statistics and models of host specialization) using the exclusivity coding (hereafter “100–0 exclusivity”). However, in the exclusivity coding, generalists and non-exclusive specialists are coded in one state (“generalists”) and thus results might be hard to interpret.

### Mega-phylogeny approach

To test dynamics of host switching, we used phylogenetic comparative methods (PCMs). For this purpose, we applied a mega-phylogeny approach using the R package “megaptera”, a pipeline for large-scale automated sequence-retrieval and alignment [44] (version available on https://github.com/heibl/megaptera). The mega-phylogeny approach aims at maximising taxon sampling integrating previous knowledge (e.g. taxonomic information, backbone trees) into the tree inference [45]. For our mega-phylogeny approach, we used a backbone guide tree based on phylogenomic analyses [24, 26, 29] to provide information for deep splits (order level), as resolving such ancient divergences can be difficult due to sequence saturation [45]. Further, mega-phylogeny approaches often lead to a high number of gaps or missing data, often more than 90% (e.g. Smith et al. [45]). To reduce the bias of missing data, we computed a reliability measure for each column of the alignment, which is then supplied to the tree inference program. In this way, uncertain regions in the alignment are down-weighted in the phylogeny inference step.

First, we used the R package “megaptera” to download all sequences for the species with decay mode and host association information from GenBank [46] (queried February 2017). We selected seven DNA regions: 18S, 28S and 5.8S rRNA (nuclear ribosomal RNA genes), genes encoding RNA polymerase b (*rpb*1, *rpb2*), translation elongation factor 1 *(tef1*), and ATP synthetase (*atp6*). We chose the rRNA regions to obtain high species numbers and the other regions for resolution of deeper nodes [47]. Only sequences of samples identified to species level were accepted.

We used single sequences where only one sequence for a particular species and DNA region was available. If multiple sequences were available, all sequences of the same DNA region and organism (putatively conspecific sequences) were aligned and a majority rule consensus sequence was calculated. In the next step, all sequences were compared to three to six Agaricomycotina reference sequences for each DNA region as a quality check (Additional file 1: Table S4). We used the R package “megaptera” to calculate the identity (proportion of nucleotides identical) and coverage (proportion of nucleotide positions in common) with the reference. Based on the coverage and identity values, thresholds can be adjusted aiming to maximize both quality and number of taxa. The default values are 0.75 for identity and 0.5 for coverage. Based on visual inspection of the alignments, we chose identity thresholds between 0.5 and 0.75 and coverage thresholds between 0.25 and 0.5 for the seven gene regions. All sequences outside these limits were discarded.

We aligned the remaining sequences for each gene region separately, using GUIDANCE2 [48, 49] with the multiple sequence alignment program MAFFT [50]. GUIDANCE2 computes a reliability score for each column based on alternative alignments produced by bootstrap guide trees and four co-optimal alignments based on each bootstrap alignment, created by the heads or tails algorithm [51]. We passed the resulting column score as character weights to the phylogeny inference program RAxML (flag -a; see additional details on phylogenetic inference below) rather than filtering the alignment using the column score, which is not recommended [52]. We used IQ-TREE version 1.5.3 with specification “-TESTMERGEONLY” [53, 54] to select a partition scheme among the gene regions. IQ-TREE found six blocks as the best partitioning scheme (merging the 5.8S rRNA and 28S rRNA into one partition; Additional file 1: Table S1). The final alignment had 37,466 sites and the proportion of gaps was 92.07% with 16,814 distinct alignment patterns.

We produced a comprehensive backbone guide tree by first assembling an order-level “genomic” based backbone tree (Additional file 1: Figure S1 A) from the literature [21, 26, 29] and then attaching all species on the order-level tips of the genomic backbone tree (Additional file 1: Figure S1 B). We performed maximum likelihood estimation, using the concatenated supermatrix of the seven DNA regions, with RAxML [55] on the CIPRES Science Gateway v.3.3 (RAxML -HPC2 on XSEDE 8.1.11) [56, 57] under the GTRGAMMA model with partitioning as described above, the GUIDANCE2 column score (flag –a) and the comprehensive backbone tree (flag –g). We subsequently conducted 1000 approximate Shimodaira–Hasegawa likelihood ratio tests (SH-aLRT branch support). SH-aLRT which are fast, accurate and robust even for larger phylogenies [58].

We estimated divergence times of the resulting phylogeny using penalized likelihood as implemented in the R function *chronos* from the R package “ape” [59]. We used two calibration points, a Late Cretaceous mushroom fossil *Archaeomarasmius legetti* [60], which bears a strong resemblance to extant Agaricales (particularly Marasmiaceae), and a Middle Eocene ectomycorrhizal fossil, which has been interpreted as a representative of Boletales [61]. We followed the strategy of Kohler et al. [26] and used the ectomycorrhizal fossil to calibrate Boletales with a stem age of 40–60 MYA and *A. legetti* to date Agaricales with a stem age range of 70–110 MYA. We also tried the approach of [24] and used 50 and 90–94 MYA as age priors, which yielded almost identical divergence time estimates (results not shown).

We applied *chronos* with three different models of substitution rate variation among branches: “relaxed”, “correlated” and “strict” and compared the model fits using ɸIC [62]. The “correlated” model had lowest ɸIC values and thus was used for further analysis. We are aware that penalized likelihood does not make use of the sequence data and does not incorporate phylogenetic uncertainty. However, algorithms that perform joint inferences of the tree and divergence times currently do not implement an option for character weights, e.g. BEAST [63] or character weights and guide tree, e.g. ExaBayes [64].

To account for phylogenetic uncertainty at nodes with low support values, we produced alternative trees based on the maximum likelihood phylogeny (Additional file 1: Figure S2). We created hard polytomies on nodes with SH-like support values < 80 based on the non-ultrametric ML tree (Additional file 1: Figure S3). We then used the function *multi2di* from the R package “ape” [59] and resolved the polytomies randomly and used *chronos* (as described above) to estimate divergence times. We repeated this 100 times and summarized the dated trees using TreeAnnotator [65] to calculate a maximum clade credibility tree (MCCT) with the node option “Common ancestor heights” (because the nodes did not share the same ancestors since polytomies were created at random). We displayed confidence intervals of the divergence time estimates as HPD (highest posterior density) for the brown rot clades and the root. Furthermore, we use the 100 ultrametric trees as input for the transition rates estimation to measure robustness of the results against phylogenetic uncertainty.

### Statistics and models of host specialization

We first tested preferences of host species among extant fungi of the two decay modes using a phylogenetic linear model in the R package “phylolm” [66]. We tested whether the number of host species (host range) differed between decay modes as a binary predictor variable. As an evolutionary model for the residual variance-covariance matrix we used the lambda model [67]. The number of host tree species was log_10_-transformed.

We modelled dynamics and pattern of host specialization evolution in white and brown rot lineages using multistate likelihood-based models. We used the function *rayDISC* from the R package “corHMM” [68], which implements a multi-state version of a continuous-time Markov model, where the Markov process is characterized by a Q-matrix. The Q matrix specifies the transition rates between the character states and hence the model of discrete character evolution. All models were based on our six-state character coding and the transition rate matrix was a 6 × 6 matrix: (1) white rot/angiosperm specialist, (2) brown rot/angiosperm specialist, (3) white rot/gymnosperm specialist, (4) brown rot/ gymnosperm specialist, (5) white rot/generalist, and (6) brown rot/generalist.

The first model allows for all transitions to occur in single steps, e.g. an angiosperm specialist can switch directly to a gymnosperm specialist without first passing through a generalist state. Further, in this model transitions between white rot and brown rot are allowed in both directions. All models allow white rot to brown rot transitions. We call this the “Uncorrelated” model, because switches between the states are not conditioned on previous states. This model may not be biologically realistic. Transitions from an angiosperm specialist to a gymnosperm specialist may require a transition first through a generalist, before passing to a gymnosperm specialist, and thus could require two “steps”. Thus, we coded further models implementing correlated (dependent) character evolution. In the second model, we prohibited transitions leading directly from one specialist to another by setting the direct transition parameters to zero. We call this the “Correlated hosts” model. Both the “Uncorrelated” and the “Correlated hosts” model allow for brown rot to white rot reversals. However, brown rot evolution is correlated with complete losses of genes encoding ligninolytic class II peroxidases (AA2) and reductions in other decay enzymes, making reversals to white rot unlikely [24]. Accordingly, we constructed a third model where we further disallowed transitions from brown rot states to white rot states. We call this the “Correlated hosts – norev” model. For the coding of the Q matrices, see Additional file 1: Figure S4.

We fitted the three models with equal rates (ER) and all rates different (ARD) and compared the fit of the models by Akaike’s information criterion (AIC) [69] from the log-likelihoods. For model selection we applied a simple root state with equal weights among the six character states (root.p = NULL). Brown rot has been shown to evolve repeatedly from white rot ancestors [70, 71], so we applied an additional root state treatment which only allows white rot as root state. Thus, after model selection we ran the final (best) model using an additional root state coding, which assumed zero probability for brown rot and equal probabilities for each of the three white rot states, and compared the models.

Another framework to estimate pattern of host evolution is the coding as three independent binary states: white rot – brown rot; angiosperm – no angiosperm; gymnosperm – no gymnosperm (e.g. using the function *corDISC*, from the R package “corHMM”). However, this model requires unobserved states (no angiosperm and no gymnosperm host). Such unobserved states may yield high rates as a methodological artefact [72]. Thus, we decided to use the multi-state implementation in the function *rayDISC*.

### Phylogenetic signal

We computed phylogenetic signal in decay mode, gymnosperm association, and the six-state character coding (as defined above). For the decay mode (binary state) we used the phylogenetic D statistic, which is calculated as the sum of sister-clade differences based on reconstructed values on all nodes of the tree [73]. The observed D is then compared against (1) a random expectation (random shuffling of trait values along the tips), and (2) a trait simulated according to a Brownian motion model of character evolution along the tree, after the values were converted to a binary according to a threshold. For the computation we used the function *phylo.d* in the R package “caper” [74] with 1000 permutations.

For the gymnosperm association we calculated two measures of phylogenetic signal: Pagel’s lambda [67] using the function *phylosig* from the R package “phytools” [75], and phylogenetic correlograms using the function *phyloCorrelogram* from the R package “phylosingal” [76]. Lambda measures the phylogenetic dependence of a trait under the assumption of a pure Brownian motion model of evolution. Lambda is a transformation (weight) of the variance-covariance matrix, if other factors than the phylogenetic history had an effect on the trait. If lambda equals 1 the model fits a Brownian motion model of evolution. Phylogenetic correlograms measure phylogenetic signal in dependence of the phylogenetic distance (that is distance in branch lengths). For a single trait, phylogenetic signal is measured as the autocorrelation (Moran’s I) based on a sequence of phylogenetic weights matrices differing in their mean (phylogenetic distance if method = “lag-norm”). We conducted 100 bootstraps for 100 points to generate a confidence interval. If the confidence interval falls below or above 0 the signal becomes significant. We rescaled the phylogeny to a tree height of 1 for this analysis.

For the six state character coding we calculated the phylogenetic signal following the method described in Bush et al. [77] (function *phylo.signal.disc*, the script is available at: https://github.com/juliema/publications/blob/master/BrueeliaMS/Maddison.Slatkin.R). A parsimony score of the discrete trait along the tree is compared to a randomized parsimony score inferred by randomizing tip states. If the parsimony score falls outside the random distribution, this indicates a higher conservation than under a random expectation.

## Results

Our core dataset consisted of 1157 fungal species, including 126 brown rot and 1031 white rot species. Based on the 90–10 specialization coding, we found 205 gymnosperms specialists, 565 angiosperm specialists and 387 generalists (for tip state frequencies, see Additional file 1: Table S2).

**Fig. 2:**
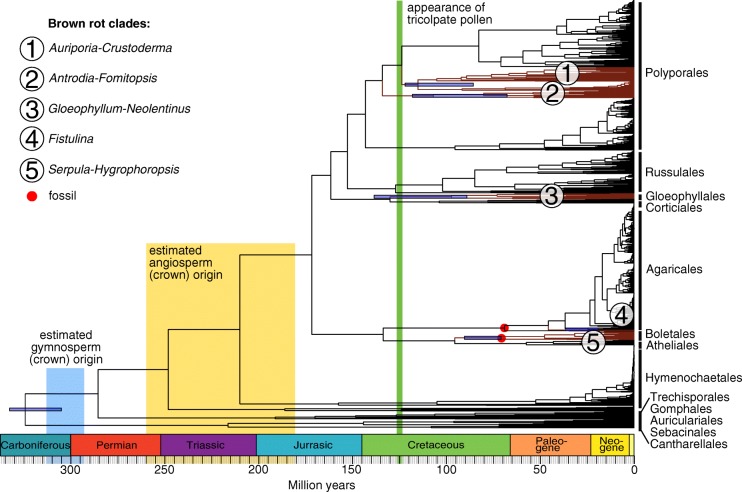
Agaricomycetes species-level dated tree of wood-decay fungi. The species-level chronogram is based on the maximum clade credibility tree (MCCT) topology, dated with two fossils indicated by black points. Clades: (1) *Auriporia-Crustoderma (Polyporales), (2) Antrodia-Fomitopsis (Polyporales), (3) Gloeophyllum-Neolentinus (Gloeophyllales), (4) Fistulina (Agaricales), (5) Serpula-Hygrophoropsis (Boletales).* Blue bars on the brown rot crown nodes indicate confidence intervals (HPD) from 100 alternative trees. For a detailed maximum likelihood phylogeny with SH support values and tip labels, see Additional file 1: Figure S3

Our time-calibrated phylogeny contains five brown rot clades (Fig. 2, all clades had SH-like support values above 90, Additional file 1: Figure S3), including two in Polyporales, one in Gloeophyllales, one in Agaricales and one in Boletales. Clade 1, the *Auriporia-Crustoderma* clade, within the Polyporales includes Laetiporaceae, Sparassidaceae, Dacryobolaceae pro parte (*Dacryobolus karstenii*), *Crustoderma* and *Pycnoporellus*. Clade 2, the *Antrodia-Fomitopsis* clade, within the Polyporales includes Fomitopsidaceae, Dacrybolaceae pro parte (*Spongiporus*, *Oligoporus*, *Postia* pro parte) and *Fibroporia gossypinum*. Clade 3, the *Gloeophyllum-Neolentinus* clade, falls within the Gloeophyllales. Clade 4, the *Fistulina* clade, falls within the Agaricales (*Fistulina pallida and F. antarctica*)*.* Clade 5, the *Serpula-Hygrophoropsis* clade, falls within the Boletales (Fig. 2).

### Phylogenetic signal

**Fig. 3:**
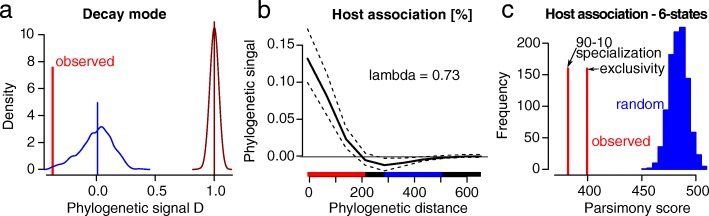
Phylogenetic signal for decay mode, and two measures of gymnosperm association. **a**) Phylogenetic signal D for decay mode (binary variable). A value smaller than 0 indicates strong conservatism. **b**) Pagel’s lambda and phylogenetic correlogram for gymnosperm association. A lambda value of 0.73 indicates non-random trait evolution which is not as conserved as Brownian motion. The phylogenetic signal increased towards the tips. Displayed is the mean phylogenetic signal with a 95% confidence interval resulting from 100 bootstraps. **c**) Phylogenetic signal C for the six-state coding. The observed value outside of the random expectation distribution indicates conservatism

For decay mode, we found a phylogenetic D value of − 0.38, which had a high probability resulting from a Brownian motion phylogenetic structure (*P* = 0.998) and a corresponding low probability resulting from a random phylogenetic structure (*P* = 0.00, Fig. 3a). We found a lambda value of 0.73 for gymnosperm association and an increasing phylogenetic signal towards the tips (Fig. 3b, red and blue lines indicates significance). For the six-state character coding, we found that the observed parsimony score was significantly smaller than under a random expectation (Fig. 3c).

### Host preferences among decay fungi

**Fig. 4:**
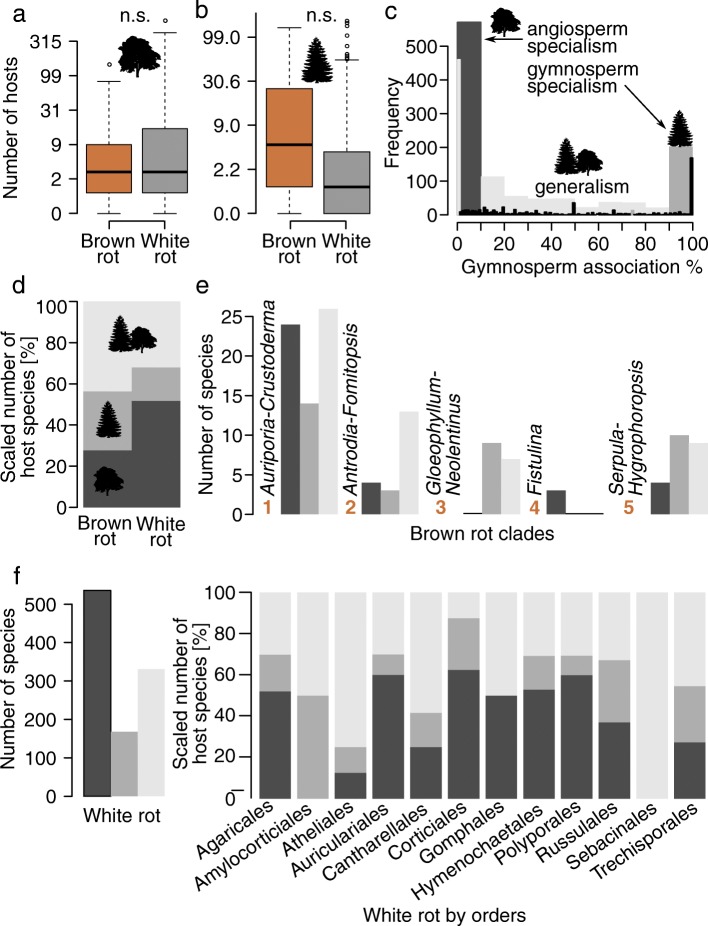
Distribution of gymnosperm association among wood-decay fungi and five major brown rot lineages within Agaricomycetes. **a**) Number of angiosperm host tree species for white and brown rot species. **b**) Number of gymnosperm host tree species for white and brown rot species. Note the log scale of the y-axis and that the values were back-transformed. Significances were inferred using phylogenetic regression (Additional file 1: Table S5). **c**) Bimodal distribution of gymnosperm association of wood-decay fungi. The six-state character coding was based on the gymnosperm association. A gymnosperm association above 90% was classified as gymnosperm specialist, below 10% as angiosperm specialist and others as generalists (“90–10 specialization”, for details see Trait data and character matrix). **d**) Scaled number of host tree species grouped by the 90–10 specialization coding. **e**) Number of species for the five observed brown rot clades (Fig. 2). **f**) Number of species for white rot fungi and scaled number of host tree species grouped by the 90–10 specialization coding. Note that Amylocorticiales, Gomphales and Sebacinales had less than five host data points. Angiosperm tree image by Michele M. Tobias (see Acknowledgements)

We assessed host preferences among extant decay fungi based on the average number of host tree species. White and brown rot fungi did not significantly differ in their average number of host tree species (phylogenetic regression, Fig. 4a, b, statistics Additional file 1: Table S5), although visible trends suggested that white rot species have a larger average host range on angiosperms (Fig. 4a), while brown rot species have a larger average host range on gymnosperms (Fig. 4b). The histogram of the gymnosperm association showed a bimodal distribution with two peaks towards the ends of the distribution, representing extremes of angiosperm vs. gymnosperm specialization (Fig. 4c). Thus, among the specialized decay fungi most occur exclusively on either angiosperm or gymnosperm hosts (Fig. 4c). Based on the gymnosperm association we found that 51% of white rot species are specialized to angiosperm hosts, whereas 27% of brown rot fungi are specialized on angiosperms (Fig. 4d). Among brown rot fungi, however, we found a higher proportion of generalists and gymnosperm specialists than in white rot fungi (Fig. 4d).

Of the five brown rot clades (Fig. 2), two consisted of mainly generalist species (Polyporales clades: *Auriporia-Crustoderma* and *Antrodia-Fomitopsis*). Two clades consist of mainly gymnosperm specialists (Gloeophyllales: *Gloeophyllum-Neolentinus*; Boletales: *Serpula-Hygrophoropsis*). One clade consists of mainly angiosperm specialists (Agaricales: *Fistulina*) (Fig. 4e). The two Polyporales clades, *Auriporia-Crustoderma* and *Antrodia-Fomitopsis,* however, also display a considerable amount of angiosperm specialists, exceeding gymnosperm specialists (Fig. 4e).

Twelve of the 14 orders in our dataset contained white rot lineages (Fig. 4f). Three of these had less than five species (Amylocorticiales, Gomphales, Sebacinales) and thus we did not interpret host associations for them. White rot species within six orders were primarily angiosperm specialists (Agaricales, Auriculariales, Corticiales, Hymenochaetales, Polyporales, Russulales) (Fig. 4f). White rot species within three orders were primarily generalists (Atheliales, Cantharellales, Trechisporales) (Fig. 4f).

### Dynamics of host switches

**Fig. 5:**
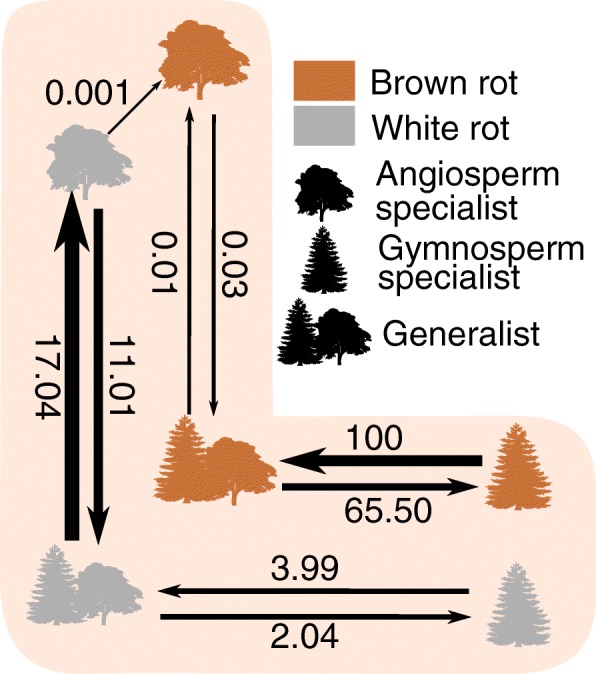
Dynamics of host specialization evolution in wood decay fungi within Agaricomycetes based on a multi-state likelihood model. Transition rates based on the best model (“Correlated hosts – norev”, Table 1) and the maximum likelihood phylogeny among six character states: white or brown rot generalist; white or brown rot angiosperm specialist and white or brown rot gymnosperm specialist. The six-state character coding was based on the gymnosperm association. A gymnosperm association above 90% was classified as gymnosperm specialist, below 10% as angiosperm specialist and others as generalists (“90–10 specialization”, for details see Trait data and character matrix). Numbers above and below arrows denote transition rates and the arrow width reflects the rate size. For rate estimates based on alternative trees and the one-genus-subsets see Additional file 1: Figure S5. Angiosperm tree image by Michele M. Tobias (see Acknowledgements)

Based on the models of discrete trait evolution describing host switching dynamics among white and brown rot fungi, we found the “Correlated hosts – norev” model, with all rates different, as the best model (model 3.2, Table 1). This model assumed host paths via a generalist state and prohibited reversal from brown rot to white rot, which is consistent with our expectation that swtiches between angiosperm and gymnosperm specialization cannot occur in a single step, and that losses of AA2s and other lignocellulolytic enzymes makes reversals from brown rot to white rot unlikely. The version of this model that specified the root state with equal weights for white rot states and zero probability for brown rot states (model 3.3) performed better than the model which assumed equal weights among all six states. We display transition rates based on model 3.3 (Fig. 5).

**Table 1:** The fit of three alternative models of host evolution among decay fungi of Agaricomycetes. The best model (shown in bold), based on Akaike weights (*w)*, was the model 3.3, which allowed only intermediate host transitions (“Correlated hosts”), no brown rot to white rot reversals (“norev”) and a root prior with equal probabilities among white rot fungi and zero probability for brown rot states (“white rot equal”). For model selection based on the exclusivity coding, see Additional file 1: Table S6

Model	-Ln L	AIC	Δ AIC	*w*
Uncorrelated, ER	− 1870.83	3743.65	1355.72	0.00
Uncorrelated, ARD	− 1180.64	2421.27	33.34	0.00
Correlated hosts, ER	− 1774.09	3550.19	1162.25	0.00
Correlated hosts, ARD	− 1183.56	2395.12	7.19	0.02
Correlated hosts – norev, ER	− 1941.71	3885.41	1497.48	0.00
Correlated hosts – norev, ARD, root = equal	−1183.66	2389.32	1.39	0.33
Correlated hosts – norev, ARD, root = white rot equal	− 1182.97	2387.93	0.00	0.65

We found disparity in rates of transitions between generalism and angiosperm specialization between the decay modes. While white rot lineages display high transition rates from generalism to angiosperm specialization, brown rot lineages display higher rates from gymnosperm specialization to generalism (Fig. 5). White rot lineages further show higher rates of transitions towards angiosperm specialization than the reverse, whereas brown rot lineages show the opposite, with higher rates from angiosperm specialization to generalism than the reverse. White and brown rot lineages both switch more frequently from gymnosperm specialization to generalism than the reverse (Fig. 5). The transition rate estimates were consistent across 100 alternative trees (Additional file 1: Figure S5 A, B). The 100 one-genus-subsets yielded consistent relative rates, but rates of white rot states were higher (especially rates from generalists to gymnosperm specialists, Additional file 1: Figure S5 A, C).

Concerning the rates of transitions from white to brown rot estimated based on the ML phylogeny, the alternative trees and one-genus-subsets did not yield a clear picture. The rate estimates based on the ML phylogeny showed one transition rate from white to brown rot angiosperm specialists (Fig. 5). The 100 alternative trees further displayed equally high rates from white to brown rot generalists (Additional file 1: Figure S5 A, B). Within the 100 alternative trees, brown rot clades were not collapsed since SH-like support values were > 90. Transition rates from white to brown rot gymnosperm specialists were either estimated as zero or very low (Fig. 5, Additional file 1: Figure S5).

## Discussion

Brown rot fungi as a whole comprise a larger proportion of gymnosperm specialists than white rot (Fig. 4d), which is consistent with Gilbertson’s observations [31]. Nevertheless, most brown rot fungi are generalists and only two of five brown rot clades display mainly gymnosperm specialists (clades *Gloeophyllum-Neolentinus* and *Serpula-Hygrophoropsis*, Fig. 4d, e). Brown rot lineages show a higher rate of switches to gymnosperm specialization than white rot fungi, but brown rot display the highest rate towards generalism. Brown rot further displayed dynamic transitions between generalism and specialization (Fig. 5). White rot fungi are highly specialized on angiosperm hosts (Figs. 4 and 5).

Gilbertson [p. 33] suggested that “85% of brown-rot polypores occur primarily on conifers”, which was the basis for later hypotheses about brown rot evolution in general [31]. Our analysis could not confirm that brown rot Polyporales occur primarily on gymnosperm hosts (Fig. 4e). We found two brown rot clades within Polyporales, of which the *Auriporia-Crustoderma* clade consists of mainly generalists and angiosperm specialists and the *Antrodia-Fomitopsis* clade of mainly generalists (Fig. 4e). Thus, neither of the two brown rot clades within the Polyporales were mainly specialized on gymnosperms (Fig. 4e). Our dataset allowed us to extend and evaluate Gilbertson’s statement for a broad range of brown rot lineages of different clades and orders. According to our analysis, only two of five brown rot clades consist of mainly gymnosperm specialists, the *Gloeophyllum-Neolentinus* (Gloeophyllales) and the *Serpula-Hygrophoropsis* (Boletales) clades (Fig. 4e). Further, the majority of brown rot fungi are generalists (Fig. 4d). Therefore, the hypothesis that brown rot fungi occur primarily on gymnosperms is not generally supported.

Based on our 90–10 specialization coding and a multi-state likelihood model of host evolution, we found that white rot fungi switched frequently between generalism and angiosperm specialism with a higher rate towards angiosperm specialism (Fig. 5). Within brown rot lineages, this pattern shifted towards frequent switches between generalism and gymnosperm specialization (Fig. 5). This suggests that brown rot evolution promoted frequent shifts to gymnosperm specialization. However, the reversal rate from gymnosperm specialism to generalism is higher, suggesting that specializations towards conifer hosts are not restrictive (Fig. 5). Hibbett and Donoghue [32], based on a much smaller dataset, inferred a correlation between brown rot and exclusive decay of conifer hosts and suggested that brown rot promotes gymnosperm specialization. However, within brown rot, transition rates between gymnosperm specialization and generalism are high in both directions, with a trend toward generalism, suggesting that specializations towards conifer hosts are not stable (Fig. 5). Our findings are robust against topological and branch lengths variation (Additional file 1: Figure S5 A, B). Further, our results are robust against different assumptions concerning reversals from white to brown rot. Transition rate estimates of the model allowing reversals and the one disallowing reversals were nearly identical (data not shown, however, AIC difference only 7.19 which is often considered as not substantially different [78]).

Further, we estimated the likelihood model of host specialization evolution based on the exclusivity coding and found that the transition rate towards gymnosperm exclusivity was higher for brown rot compared with white rot lineages (Additional file 1: Figure S6). This finding is consistent with the 90–10 specialization coding (Fig. 5). Within the exclusivity model we found overall higher rates from host exclusivity to generalism (Fig. 5, Additional file 1: Figure S6). However, the stringency of this coding scheme may overestimate the number of generalist taxa, as species found at extremely high rates on a single host species (e.g. > 90%, but less than 100%) are still coded as generalists. Thus, rates towards “generalists” are probably overestimated in this coding scheme. Therefore, interpretations from the exclusivity model should be made with caution. For a more detailed picture, further analysis should thus include three states of host association, separating generalism, non-exclusive specialization and exclusivity and treat non-exclusive specialization as an intermediate state.

Based on our time-calibrated mega-phylogeny approach, we found that most lineages within Agaricomycetes radiated after the origins of gymnosperms and angiosperms (Fig. 2). Our estimates for branching times are highly consistent with chronograms of previous studies with more limited species sampling, but more genomic information. Floudas et al. [24] for example found a mean age of 290 million years for the crown node of Agaricomycetes, which is consistent with our estimate of 282 million years (Additional file 1: Figure S7). Smith et al. [79] used an uncorrelated relaxed molecular clock analysis to date a comprehensive plant tree of life and found mean crown origins of 301 million years for gymnosperms and 217 million years for angiosperms, respectively. Many of the large clades within Agaricomycetes originated before, but diversified after the angiosperm and gymnosperm origins (Fig. 2). The estimated timing of origin of the fungal and plant groups is consistent with our inference that transitions from white rot to brown rot occurred among angiosperm specialists (Fig. 5) or possibly generalists (Additional file 1: Figure S5). Relative transition rates in white rot fungi suggest a pattern of transition away from gymnosperm specialization and towards generalism, followed by relatively higher rates of angiosperm specialization (Fig. 5). This pattern away from gymnosperm specialization and towards angiosperm specialization among white rot is consistent with the relatively high percentage of white rot angiosperm specialists we observed (Fig. 4). Thus, it is plausible that the radiation of angiosperms created new niches for wood decayers and promoted diversification of white rot fungi.

## Conclusion

Our models of host evolution suggest that angiosperms may have served as a new mega-niche, which was exploited particularly well by white rot fungi leading to high specialization rates. Brown rot lineages switched more frequently towards generalism, suggesting that brown rot fungi were limited in exploiting angiosperm resources. Whether this limitation on the part of brown rot in exploiting angiosperm resources is directly related to the loss in copy number of decay-related genes [26] seems plausible, but remains to be tested by future studies. Moreover, host shifts may be identifiable at the enzymatic level, if expression patterns for genes coding for key decay enzymes differ between clades with different host specializations. Such studies represent exciting future possibilities in this system, and may elucidate the underlying molecular mechanisms controlling decay mode shifts.

## Additional file

Additional file 1: rusda: an R interface to the United States Department of Agriculture’s Fungus-Host Distribution Database. **Figure S1.** Genomic phylogenetic tree compiled from Floudas et al. 2012; Kohler et al. 2015; Nagy et al. 2015 used as the backbone for the comprehensive guide tree for the RAxML tree inference. **Figure S2.** Maximum Likelihood phylogeny of the Agaricomycetes with color coding for 14 orders. **Figure S3.** Phylogeny with SH support values. **Figure S4.** Transition rates between the states in 6 × 6 Q-matrices with six states: (1) white rot/angiosperm specialist, (2) brown rot/angiosperm specialist, (3) white rot/gymnosperm specialist, (4) brown rot/ gymnosperm specialist, (5) white rot/generalist, and (6) brown rot/generalist. **Figure S5.** Transition rates among six character states based on a maximum likelihood (ML) phylogeny, 100 alternative trees and the one-genus-subset (100 times bootstrapped). **Figure S6.** Dynamics of host specialization in wood decay fungi within the Agaricomycetes based on the 100–0 exclusivity coding. **Figure S7.** Branching times for backbone of time-dated Agaricomycetes phylogeny. Figure S8 Overview of query results using R package “rusda” based on the Fungus-Host Distribution Database (FHDD) using 29,591 Dikarya and 105,350 Spermatophyta species as input. **Table S1.** Best partition scheme found by IQ-Tree [11, 12]. **Table S2.** Tip state frequencies of white and brown rot specialization based on different thresholds of host association [%]. **Table S3.** Re-classification of taxonomic orders based on Binder et al., 2005; Hibbett et al., 2014; Larsson, Larsson, & Kõljalg, 2004. **Table S4.** Table of reference species and association numbers from NCBI. Table S5 Phylogenetic and normal linear regression on the number of angio- and gymnosperm hosts between white and brown rot species. **Table S6.** The fit of three alternative models of host association evolution in white and brown rot lineages of Agaricomycetes based on the exclusivity coding (100–0). (DOCX 22788 kb)

Additional file 2: The alignment used in maximum likelihood analyses. (FAS 43500 kb)

Additional file 3: Newick file for the maximum likelihood phylogeny of the decay fungi of this study. (TRE 56.6 kb)

Additional file 4: Newick file for the comprehensive guide tree used for the maximum likelihood phylogeny. (TRE 27.5 kb)

Additional file 5: Partition scheme for the alignment. The best partition scheme was determined using IQ-Tree, and is listed here. (TXT 153 bytes)

Additional file 6: Nexus file for the 100 alternative phylogenetic trees of the decay fungi of this study. (NEXUS 36100 kb)

Additional file 7: GUIDANCE column reliability score for the maximum likelihood phylogeny. (TXT 88.5 kb)

Additional file 8: Data sheet with host associations and decay mode. (XLSX 113 kb)

## References

[1] McCarl BA, Metting FB, Rice C (2007). Soil carbon sequestration. Clim Change.

[2] Siegenthaler U, Sarmiento JL (1993). Atmospheric carbon dioxide and the ocean. Nature.

[3] Horwath W. 12 – carbon cycling and formation of soil organic matter. Soil Microbiol Ecol Biochem. 2007:303–39.

[4] Welker C, Balasubramanian V, Petti C, Rai K, DeBolt S, Mendu V (2015). Engineering plant biomass lignin content and composition for biofuels and bioproducts. Energies.

[5] Lundell TK, Mäkelä MR, Hildén K (2010). Lignin-modifying enzymes in filamentous basidiomycetes--ecological, functional and phylogenetic review. J Basic Microbiol.

[6] Medie FM, Davies GJ, Drancourt M, Henrissat B (2012). Genome analyses highlight the different biological roles of cellulases. Nat Rev Microbiol.

[7] Sjöstrom E, Wood Chemistry: Fundamentals and Applications, Academic Press, New York, 1981.

[8] Bugg TDH, Ahmad M, Hardiman EM, Rahmanpour R (2011). Pathways for degradation of lignin in bacteria and fungi. Nat Prod Rep.

[9] Heimann M, Reichstein M (2008). Terrestrial ecosystem carbon dynamics and climate feedbacks. Nature.

[10] Harley JL. Fungi in ecosystems. J Appl Ecol JSTOR. 1971:627–42.

[11] Beech E, Rivers M, Oldfield S, Smith PP (2017). GlobalTreeSearch: the first complete global database of tree species and country distributions. J Sustain For.

[12] Thakur VK, Thakur MK. Processing and characterization of natural cellulose fibers/thermoset polymer composites. Carbohydr Polym. 2014:102–17. 10.1016/j.carbpol.2014.03.039 24815407

[13] Cornwell WK, Cornelissen JHC, Allison SD, Bauhus J, Eggleton P, Preston CM (2009). Plant traits and wood fates across the globe: rotted, burned, or consumed?. Glob Chang Biol.

[14] Pichersky E, Gang DR. Genetics and biochemistry of secondary metabolites in plants: an evolutionary perspective. Trends Plant Sci. 2000:439–45. 10.1016/s1360-1385(00)01741-6 11044721

[15] Pallardy SG. Physiology of Woody plants. Physiol Woody Plants. 2008;

[16] Wagenführ R, Scheiber C. Holzatlas. Fachbuchverlag Leipzig; 2007.

[17] Worrall JJ, Anagnost SE, Zabel RA. Comparison of wood decay among diverse lignicolous fungi. Mycologia JSTOR. 1997:199–219.

[18] Blanchette RA (1991). Delignification by wood-decay fungi. Annu. Rev. Phytopathol.

[19] Lundell TK, Mäkelä MR, de Vries RP, Hildén KS (2014). Genomics, lifestyles and future prospects of wood-decay and litter-decomposing basidiomycota. Adv Bot Res.

[20] Schneider T, Keiblinger KM, Schmid E, Sterflinger-Gleixner K, Ellersdorfer G, Roschitzki B (2012). Who is who in litter decomposition? Metaproteomics reveals major microbial players and their biogeochemical functions. ISME J.

[21] Riley R, Salamov AA, Brown DW, Nagy LG, Floudas D, Held BW (2014). Extensive sampling of basidiomycete genomes demonstrates inadequacy of the white-rot/brown-rot paradigm for wood decay fungi. Proc Natl Acad Sci U S A.

[22] Rineau F, Shah F, Smits MM, Persson P, Johansson T, Carleer R (2013). Carbon availability triggers the decomposition of plant litter and assimilation of nitrogen by an ectomycorrhizal fungus. ISME J.

[23] Maijala P, Fagerstedt KV, Raudaskoski M (1991). Detection of extracellular cellulolytic and proteolytic activity in ectomycorrhizal fungi and Heterobasidion annosum (Fr.) Bref. New Phytol.

[24] Floudas D, Binder M, Riley R, Barry K, Blanchette RA, Henrissat B (2012). The Paleozoic origin of enzymatic lignin decomposition reconstructed from 31 fungal genomes. Science.

[25] Nagy LG, Ohm RA, Kovács GM, Floudas D, Riley R, Gácser A, et al. Latent homology and convergent regulatory evolution underlies the repeated emergence of yeasts. Nat Commun. 2014;5 10.1038/ncomms5471 25034666

[26] Kohler A, Kuo A, Nagy LG, Morin E, Barry KW, Buscot F (2015). Convergent losses of decay mechanisms and rapid turnover of symbiosis genes in mycorrhizal mutualists. Nat Genet.

[27] Lombard V, Golaconda Ramulu H, Drula E, Coutinho PM, Henrissat B (2014). The carbohydrate-active enzymes database (CAZy) in 2013. Nucleic Acids Res.

[28] Hofrichter M, Ullrich R, Pecyna MJ, Liers C, Lundell T. New and classic families of secreted fungal heme peroxidases. Appl Microbiol Biotechnol. 2010:871–97. 10.1007/s00253-010-2633-0 20495915

[29] Nagy LG, Riley R, Bergmann, PJ Krizsán, K, Martin FM, Grigoriev I V, Cullen D, Hibbett DS. Genetic bases of fungal white rot wood decay predicted by phylogenomic analysis of correlated gene-phenotype evolution. Mol Biol Evol. 2016;34:35–44. 10.1093/molbev/msw238 27834665

[30] Burdsall HH, Blackwell M, Nakasone KK (2012). Robert Lee Gilbertson, 1925--2011. Mycologia Mycol Soc Am.

[31] Gilbertson RL (1980). Wood-rotting fungi of North America. Mycologia.

[32] Hibbett DS, Donoghue MJ (2001). Analysis of character correlations among wood decay mechanisms, mating systems, and substrate ranges in homobasidiomycetes. Syst Biol.

[33] Farr DF, Rossman AY, Palm ME, McCray EB. Fungal databases, systematic mycology and microbiology laboratory [Internet]. ARS, USDA. 2012. Available from: http://nt.ars-grin.gov/fungaldatabases/

[34] Tedersoo L, Bahram M, Polme S, Koljalg U, Yorou NS, Wijesundera R (2014). Global diversity and geography of soil fungi. Science.

[35] Kim SY, Park SY, Ko KS, Jung HS (2003). Phylogenetic analysis of Antrodia and related taxa based on partial mitochondrial SSU rDNA sequences. Antonie Van Leeuwenhoek.

[36] Molina FI, Shen P, Jong SC, Orikono K (1992). Molecular evidence supports the separation of Lentinula edodes from Lentinus and related genera. Can J Bot Can Bot.

[37] Rathod MM. Taxonomic studies on the Daedaloid and Hexagonoid Polypores form the Forest of western Maharasta. Recent Res Sci Technol. 2011;3

[38] Barrasa JM, Esteve-Raventós F, Dähncke RM (2006). Clitocybula canariensis (Tricholomataceae), a new brown-rot fungus from the Canary Islands (Spain). Fungal Divers.

[39] Wesenberg D, Buchon F, Agathos SN (2002). Degradation of dye-containing textile effluent by the agaric white-rot fungus Clitocybula dusenii. Biotechnol Lett.

[40] Gilbertson RL, Martin KJ, Lindsey JP (1974). Annotated check list and host index for Arizona wood-rotting fungi. College of Agriculture.

[41] Petersen RH. Gloeomucro and a note on Physalacria concinna. Mycologia. 1980:301–11.

[42] Heibl C. The megaptera package: Large phylogenetic dataset assembly in R. R package version 1.0–25. 2014.

[43] FitzJohn RG, Pennel MW, Zanne AE, Stevens PF, Tank DC, Cornwell WK. How much of the world is woody? J Ecol. 2014;102:1266–72.

[44] Heibl C. The megapera package: Large phylogenetic dataset assembly in R. Modern Phylogenetic Comparative Methods and their application in evolutionary biology. Seville, Spain,. 11–15.11.2014. 2014.

[45] Smith SA, Beaulieu JM, Donoghue MJ (2009). Mega-phylogeny approach for comparative biology: an alternative to supertree and supermatrix approaches. BMC Evol Biol.

[46] Benson DA, Cavanaugh M, Clark K, Karsch-Mizrachi I, Lipman DJ, Ostell J, Sayers EW. GenBank. Nucleic Acids Res. 2012;41:D36–D42. 10.1093/nar/gks1195 PMC3531190 23193287

[47] Binder M, Justo A, Riley R, Salamov A, Lopez-Giraldez F, Sjokvist E (2013). Phylogenetic and phylogenomic overview of the Polyporales. Mycologia.

[48] Sela I, Ashkenazy H, Katoh K, Pupko T (2015). GUIDANCE2: accurate detection of unreliable alignment regions accounting for the uncertainty of multiple parameters. Nucleic Acids Res.

[49] Penn O, Privman E, Landan G, Graur D, Pupko T (2010). An alignment confidence score capturing robustness to guide tree uncertainty. Mol Biol Evol.

[50] Katoh K, Standley DM (2013). MAFFT multiple sequence alignment software version 7: improvements in performance and usability. Mol Biol Evol.

[51] Landan G, Graur D (2008). Local reliability measures from sets of co-optimal multiple sequence alignments. Pacific Symp Biocomput.

[52] Tan G, Muffato M, Ledergerber C, Herrero J, Goldman N, Gil M (2015). Current methods for automated filtering of multiple sequence alignments frequently worsen single-gene phylogenetic inference. Syst Biol.

[53] Nguyen LT, Schmidt HA, Von Haeseler A, Minh BQ (2015). IQ-TREE: a fast and effective stochastic algorithm for estimating maximum-likelihood phylogenies. Mol Biol Evol.

[54] Chernomor O, Von Haeseler A, Minh BQ (2016). Terrace aware data structure for Phylogenomic inference from Supermatrices. Syst Biol.

[55] Stamatakis A, Hoover P, Rougemont J (2008). A rapid bootstrap algorithm for the RAxML web servers. Syst. Biol.

[56] Miller MA, Pfeiffer W, Schwartz T (2010). Creating the CIPRES science gateway for inference of large phylogenetic trees. Gatew Comput Environ Work.

[57] Miller MA, Pfeiffer W, Schwartz T. The CIPRES science gateway: a community resource for phylogenetic analyses. Proc. 2011 TeraGrid Conf. Extrem. Digit. Discov. 2011. p. 41.

[58] Anisimova M, Gil M, Dufayard J-F, Dessimoz C, Gascuel O (2011). Survey of branch support methods demonstrates accuracy, power, and robustness of fast likelihood-based approximation schemes. Syst. Biol.

[59] Paradis E, Claude J, Strimmer K. APE: Analyses of phylogenetics and evolution in R language. Bioinformatics. 2004;20:289–90. 10.1093/bioinformatics/btg412 14734327

[60] Hibbett D, Grimaldi D, Donoghue M (1997). Fossil mushrooms from Miocene and cretaceous ambers and the evolution of Homobasidiomycetes. Am. J. Bot.

[61] LePage BA, Currah RS, Stockey AR, Rothwell GW (1997). Fossil ectomycorrhizae from the middle Eocene. Am J Bot.

[62] Paradis E (2013). Molecular dating of phylogenies by likelihood methods: a comparison of models and a new information criterion. Mol Phylogenet Evol.

[63] Drummond AJ, Bouckaert RR. Bayesian evolutionary analysis with BEAST 2. Bayesian Evol. Anal. with BEAST. 2015;249.

[64] Aberer AJ, Kobert K, ExaBayes SA (2014). Massively parallel Bayesian tree inference for the whole-genome era. Mol Biol Evol.

[65] Drummond AJ, Suchard MA, Xie D, Rambaut A (2012). Bayesian phylogenetics with BEAUti and the BEAST 1.7. Mol. Biol. Evol.

[66] Ho LST, Ané C (2014). A linear-time algorithm for Gaussian and non-Gaussian trait evolution models. Syst Biol.

[67] Pagel M (1999). Inferring the historical patterns of biological evolution. Nature.

[68] Beaulieu JM, Oliver JC, O’Meara B. Package `corHMM’. 2014.

[69] Akaike H (1974). A new look at the statistical model identification. IEEE Trans Autom Control.

[70] Nagy LG, Floudas D, Riley R, Barry K, Grigoriev IV, Hibbett DS. Diversification of wood decay systems in early evolution of Agaricomycotina. Phytopathology. 2013:181.

[71] Nagy LG, Riley R, Tritt A, Adam C, Daum C, Floudas D, et. al. Comparative Genomics of Early-Diverging Mushroom-Forming Fungi Provides Insights into the Origins of Lignocellulose Decay Capabilities. Mol. Biol. Evol. 2015;33:959–70. 10.1093/molbev/msv337 26659563

[72] Beaulieu JM, Donoghue MJ (2013). Fruit evolution and diversification in Campanulid angiosperms. Evolution.

[73] Fritz SA, Purvis A (2010). Selectivity in mammalian extinction risk and threat types: a new measure of phylogenetic signal strength in binary traits. Conserv Biol.

[74] Orme D. The caper package : comparative analysis of phylogenetics and evolution in R. R Packag version 0.5, 2. 2013;1–36.

[75] Revell LJ (2012). phytools: An R package for phylogenetic comparative biology (and other things). Methods Ecol. Evol.

[76] Keck F, Rimet F, Bouchez A, Franc A (2016). Phylosignal: an R package to measure, test, and explore the phylogenetic signal. Ecol. Evol.

[77] Bush SE, Weckstein JD, Gustafsson DR, Allen J, DiBlasi E, Shreve SM (2016). Unlocking the black box of feather louse diversity: a molecular phylogeny of the hyper-diverse genus Brueelia. Mol. Phylogenet. Evol.

[78] Burnham KP, Anderson DR. Model selection and multimodel inference: a practical information-theoretic approach. Springer Science & Business Media, 2003.

[79] Smith SA, Beaulieu JM, Donoghue MJ (2010). An uncorrelated relaxed-clock analysis suggests an earlier origin for flowering plants. Proc Natl Acad Sci.

