# Supplementary material for: Evolutionary dynamics of host specialization in wood-decay fungi
Source: BMC Evol Biol. 2018 Aug 3;18:119. doi: 10.1186/s12862-018-1229-7 (PMC6091043; doi:10.1186/s12862-018-1229-7)
Supplement: Supplementary file 1 — rusda: an R interface to the United States Department of Agriculture’s Fungus-Host Distribution Database. Figure S1. Genomic phylogenetic tree compiled from Floudas et al. 2012; Kohler et al. 2015; Nagy et al. 2015 used as the backbone for the comprehensive guide tree for the RAxML tree inference. Figure S2. Maximum Likelihood phylogeny of the Agaricomycetes with color coding for 14 orders. Figure S3. Phylogeny with SH support values. Figure S4. Transition rates between the states in 6 × 6 Q-matrices with six states: (1) white rot/angiosperm specialist, (2) brown rot/angiosperm specialist, (3) white rot/gymnosperm specialist, (4) brown rot/ gymnosperm specialist, (5) white rot/generalist, and (6) brown rot/generalist. Figure S5. Transition rates among six character states based on a maximum likelihood (ML) phylogeny, 100 alternative trees and the one-genus-subset (100 times bootstrapped). Figure S6. Dynamics of host specialization in wood decay fungi within the Agaricomycetes based on the 100–0 exclusivity coding. Figure S7. Branching times for backbone of time-dated Agaricomycetes phylogeny. Figure S8 Overview of query results using R package “rusda” based on the Fungus-Host Distribution Database (FHDD) using 29,591 Dikarya and 105,350 Spermatophyta species as input. Table S1. Best partition scheme found by IQ-Tree [11, 12]. Table S2. Tip state frequencies of white and brown rot specialization based on different thresholds of host association [%]. Table S3. Re-classification of taxonomic orders based on Binder et al., 2005; Hibbett et al., 2014; Larsson, Larsson, & Kõljalg, 2004. Table S4. Table of reference species and association numbers from NCBI. Table S5 Phylogenetic and normal linear regression on the number of angio- and gymnosperm hosts between white and brown rot species. Table S6. The fit of three alternative models of host association evolution in white and brown rot lineages of Agaricomycetes based on the exclusivity coding (100–0). (DOCX 22788 kb) [file 12862_2018_1229_MOESM1_ESM.docx]

**Supplement**

Additional file

**Supplementary Text**

**rusda: an R interface to the United States Department of Agriculture`s Fungus-Host Distribution Database**

*Background*

The United States Department of Agriculture (USDA) has made great effort to compile global fungus-host combinations data. The USDA thus created the Fungus-Host-Distribution Database (FHDD) to store this data and enable public access. However, data from this database is not easily available, requiring users to download species information by hand. This procedure is very time consuming. If large amounts of data are downloaded, error might occur while compilation of many single files. The risk of such error increases with the number of taxa required, which increased in recent years. Comparative analysis often require hundreds or even thousands of species [1–3]. A fast and open access to these data may provide basic research on the ecology and evolution of fungus-host associations.

To this end, the first author (Franz-S. Krah) developed an R package allowing rapid and automated access to a large global database of fungus-host combinations by the FHDD. This database involves more than 300.000 unique fungus-host combinations [4]. However, the web interface of the database makes data analysis difficult, limiting the use of this database. The aim of the R package “rusda” is to make the data contained in the FHDD readily accessible from R, an open source statistical programming environment.

*Core package function*

Here, the core functions of the “rusda” package will be presented. The R package “rusda” is available on GitHub via the rOpenSci collective. rOpenSci is a research community committed to make scientific data retrieval open and reproducible using shared data and reusable software. The package can be downloaded using the following code:

# Install R package “rusda”

install.packages(“devtools”)

devtools::install_github(“ropensci/rusda”)

library(“rusda”)

*Querying the database*

The USDA Fungus-Host Distribution Database (FHDD) contains data on host (plants)-fungus combinations. Further it contains a Nomenclature and a Literature database, which are also accessible via “rusda”. Within the FHDD only published literature was used as data basis. Besides the FHDD there is the Specimens Database (SD) which refers to deposited specimens in the U.S. National Fungus Collections (BPI). The core function of “rusda” is the function *associations*. Using *associations* queries can be made to find all plant associations of a known fungus (spec_type = “fungus”) or to find all fungal associations of a known plant host (spec_type = “plant”). When querying the FHDD, the user can input species or genus names or any higher taxon.

# The following example code queries fungus-host associations for the fungal species

# *Magnaporthe oryzae*

magna.hosts <- associations(x = “Magnaporthe oryzae”, spec_type = “fungus”, database = “FH”)

# The following example code queries fungus-host associations for all species within the

# family Cucurbitaceae

curc.fungi <- associations(x = “Cucurbitaceae”, spec_type = “plant”, database = “FH”)

The argument “database” specifies whether the FHDD or the SD or both should be queried. Further arguments allow clean steps, verbosity of the function or if synonyms of the input should be incorporated in the search. The default is with a cleaning step, which eliminates non-Linnean species names from the results list. Further, by default synonyms are queried to increase query success. The resulting structure of the “curc.fungi” object is a list of three objects. The first lists synonyms of the input names. The second is a data table with the input species and the queried combinations and the country of the record. The third is a data table with the input species and the study identifier number (ID). The IDs can be directly used as input to the function “getStudy” to obtain the full citation.

*Exploration of the Fungus-Host Distribution Database (FHDD)*

The website of the FHDD website does not yield detailed information about the taxonomic distribution of the data stored in the database. To assess the general usability of the FHDD database, and therefore the R package “rusda”, we used two species input datasets. We therefore downloaded two taxonomies for all species of Dikarya and Spermatophyta from the NCBI taxonomy (function *stepA* from the R package “megaptera” [5]). The resulting taxonomies exhibit Linnean species names only, which were used as input for the function *associations* of “rusda”. The length of the input species sets were 29,591 for Dikarya and 105,350 for Spermatophyta. The function *associations* queries the related fungal (for plant input) or plant (for fungus input) associations for each species from the datasets. Note that the function *associations* also accepts names of higher taxa as input, however we the plant and fungus input names were too long to download within single sessions. Thus we downloaded associations in batches of 1000 species.

We used both databases and considered results for synonyms of the input species and queried fungus-host associations for the two input species sets. We found data for 11,146 Dikarya (37.6 % of input) and 17,345 for Spermatophyta (16.5 % of input). A total of 268,752 (90 % of website information) combinations were found for Dikarya in the FHD. The discrepancy of 10 % can be explained by the cleaning step of our algorithm. It deletes records that are no valid species or genera names (e.g. ”wood, submerged”) since usually the user is only interested in taxonomic data. Another reason is that we did not query for non-fungal lineages like Oomycetes (e.g. *Phytophthora*)*,* which are also present (as pathogens) in the database. We found 87,232 unique combinations for the Spermatophyta input set, which could not be evaluated due to lacking information on the website (Farr et al. 2012).

We then matched the retrieved hosts and fungi against the plant and fungus taxonomies to compute the number if species with host or fungus information respectively. Therefore, we compared the number of species with information based on the FHDD compared with the total species number for a given order in the NCBI taxonomy. The results are summarized in the Fig. S8. We hope that this figure is a useful tool for scientists interested in host associations.

Here we want to point out that of the 13 investigated orders (bold) in this study, six orders had more or ca. 50% of species with host information. Among those are the orders with most representation in our dataset: Polyporales, Agaricales, Hymenochaetales. The other groups had below 50% of species with host information (Fig. S8). On the plant side we found the two orders with the majority of woody plants in the temperate zone, Fagales and Pinales, have a very good representation in the database. All plant species within the NCBI taxonomy (with valid Genus and species names) have at least 1 host association information. However, there are other mainly-woody orders, that are not very well represented: e.g. Ericales. This overview shows the need for further recordings and assembly of fungus-host associations in the FHDD (Fig. S8).

**Figures and Tables**

**Fig. S1** Genomic phylogenetic tree compiled from Floudas et al. 2012; Kohler et al. 2015; Nagy et al. 2015 used as the backbone for the comprehensive guide tree for the RAxML tree inference. Dacrymycetales served as outgroup. A) Backbone of the guide tree. B) Comprehensive guide tree, which is the backbone guide tree with species attached based on their systematic order classification with zero branch lengths.


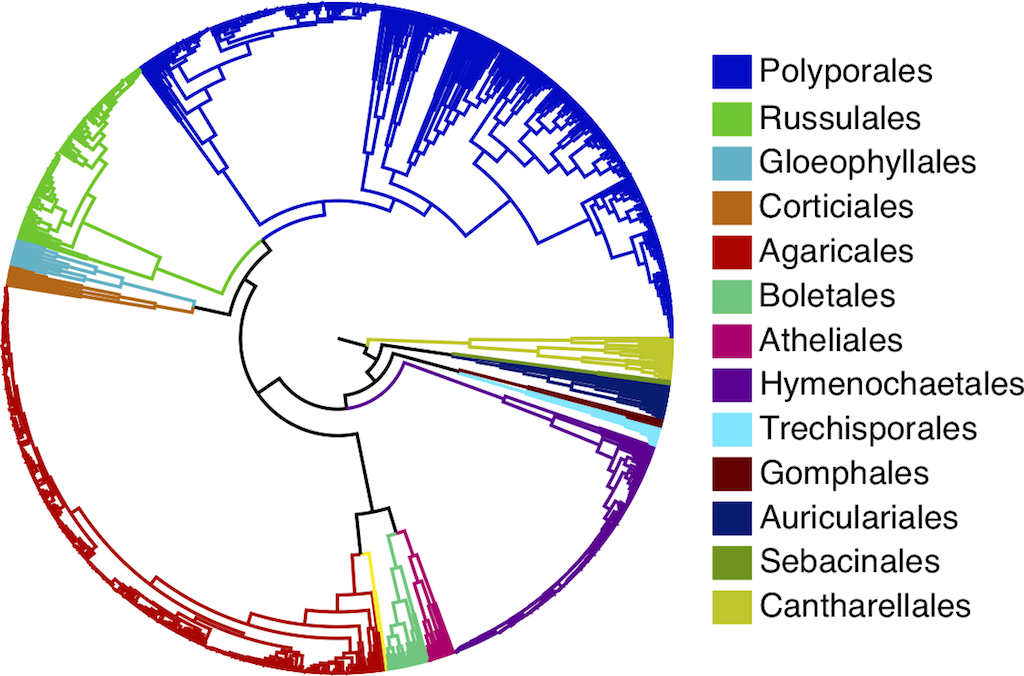


**Fig. S2** Maximum Likelihood phylogeny of the Agaricomycetes with color coding for 14 orders. The largest orders in terms of tips are Polyporales, Agaricales, Hymenochaetales and Russulales.

**Fig. S3 Phylogeny with SH support values**. A zoom-able version with species names and Shimodaira–Hasegawa approximate likelihood ratio test support values (SH-aLRT branch support).

**
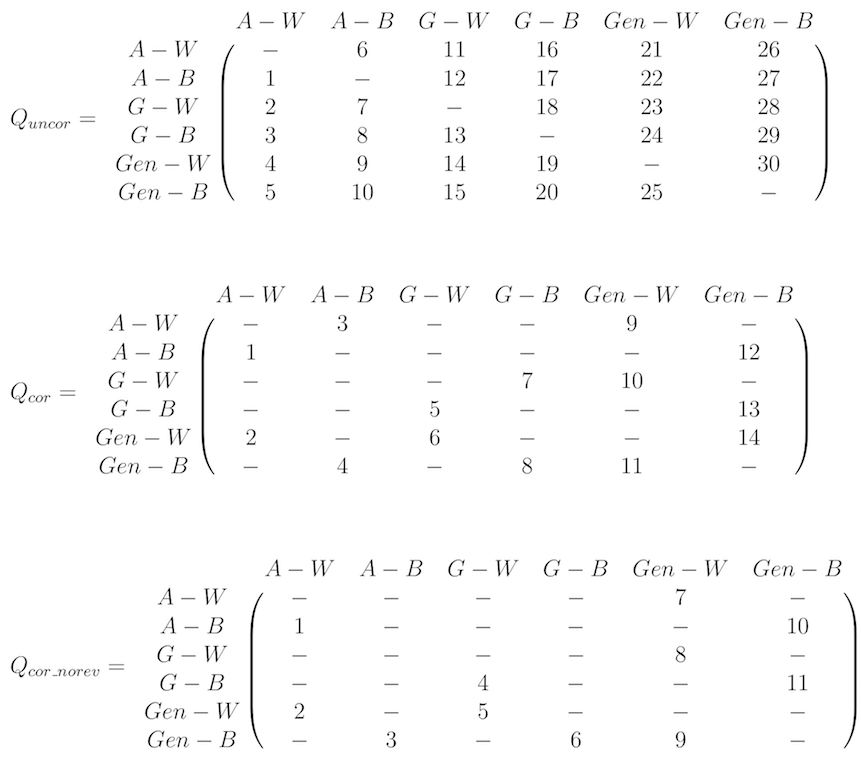
**

**Fig. S4** Transition rates between the states in 6×6 Q-matrices with six states: (1) white rot/angiosperm specialist, (2) brown rot/angiosperm specialist, (3) white rot/gymnosperm specialist, (4) brown rot/ gymnosperm specialist, (5) white rot/generalist, and (6) brown rot/generalist. The first model displays is the “Uncorrelated” model (Q_uncor_) with 30 parameters (rates). The second model is the “Correlated hosts” model (Q_cor_), which forces host shifts to pass through intermediate states (e.g. A-W to Gen-W to G-W, instead of A-W to G-W). The third model displays is the “Correlated hosts” model, which additionally does not allow reversals from white rot brown rot (Q_cor_norev_). Numbers are indices of the rates.

**
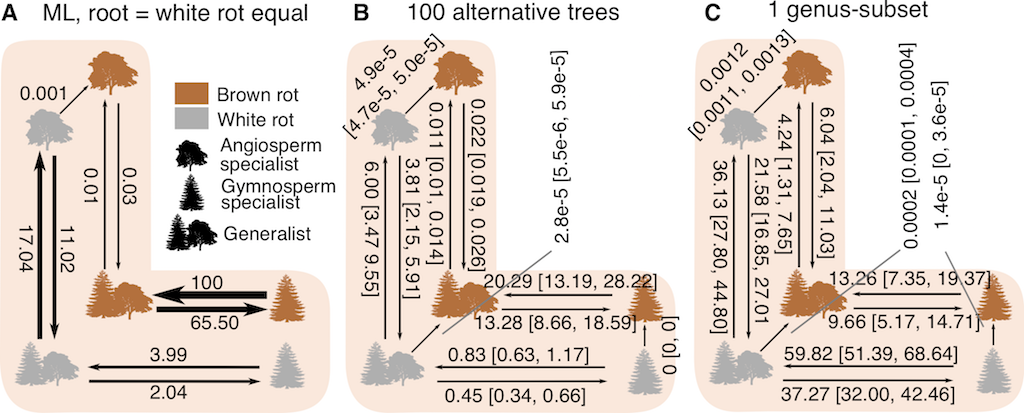
**

**Fig. S5** Transition rates among six character states based on a maximum likelihood (ML) phylogeny, 100 alternative trees and the one-genus-subset (100 times bootstrapped). Transition rates based on the 90-10 (%) specialization thresholds with root state set to equal probabilities among the white rot states and zero probability for brown rot states (Table 1). Numbers above and below arrows denote transition rates and the arrow width reflects the rate size. A) Same as Fig. 4. B) Transition rates based on 100 alternative trees with the bootstrapped 95% confidence interval (function *smean.cl.boot* from theR package “Hmisc”, Harrell Jr et al. 2017). Rates are consistent with the ML rates (A) in terms of relative size. 100 trees were produced by creating hard polytomies on nodes below a SH support threshold of 80. The polytomies were then resolved 100 times and divergence time (function *chronos*) was estimated (for details see method section). C) We extended species decay mode information to the genus, where further data was missing. Thus we bootstrapped the full dataset and phylogeny to a single species per genus and estimated transition rates. Although relative rates (especially between rates and their reversal rates) remain consistent, white rot transition rates are much higher in total. This might be explained by the extreme imbalance of the number of genera (brown rot: 40 genera, white rot: 232 genera) together with a very small phylogeny (N_tip_ = 272).


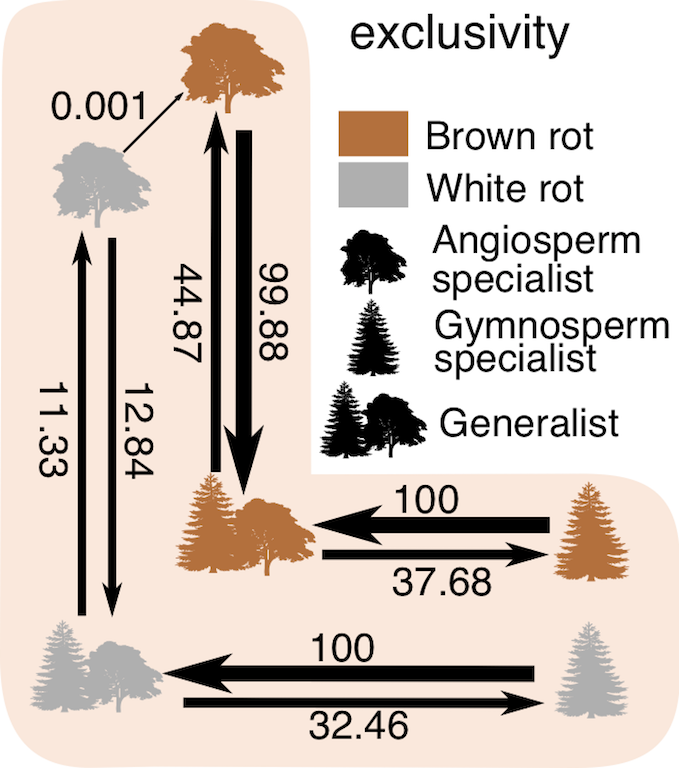


**Fig. S6** Dynamics of host specialization in wood decay fungi within the Agaricomycetes based on the **100-0 exclusivity** coding. Transition rates were further based on the (best) model 3.3 (Table 1) and the maximum likelihood phylogeny. We used a multi-state model of host specialization evolution with six character states: white or brown rot generalist; white or brown rot angiosperm specialist and white or brown rot gymnosperm specialist. Rates towards generalism are likely overestimations because “generalism” in this model incorporates generalists as well as specialists. Numbers above and below arrows denote transition rates and the arrow width reflects the rate size.

**
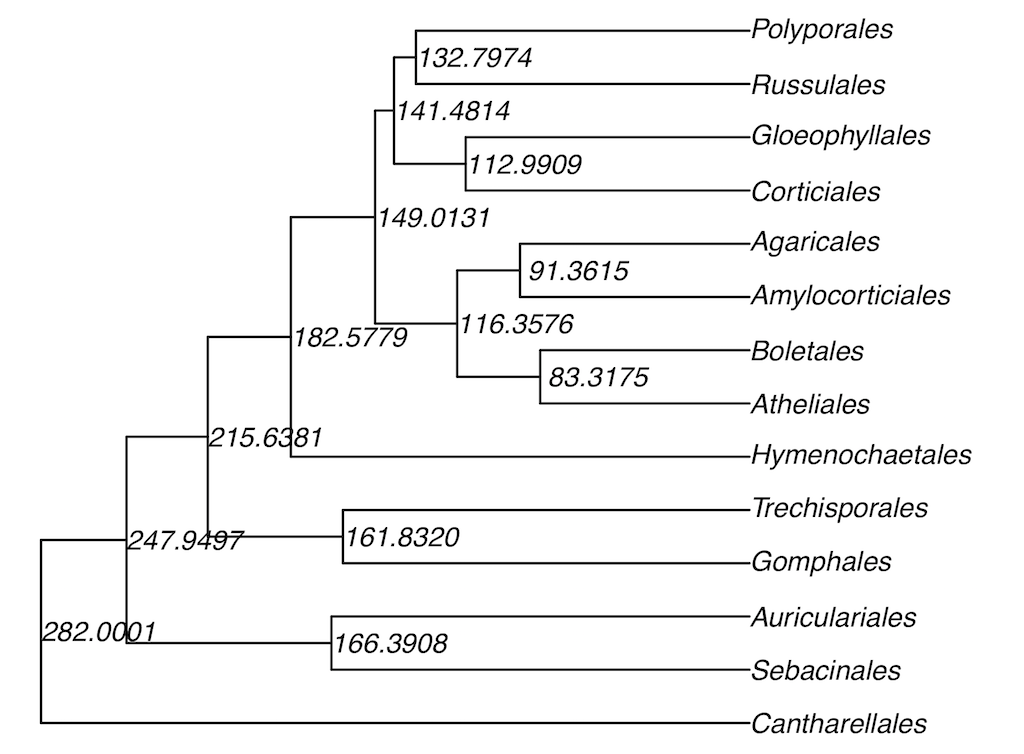
**

**Fig. S7 Branching times for backbone of time-dated Agaricomycetes phylogeny.** Branching times are based on the maximum likelihood phylogeny. Here the full phylogeny was reduced to order level to display backbone crown age estimates. The root was dated with an age of 282 million years, which fits well to previous estimates for the Agaricomycetes [6,8].


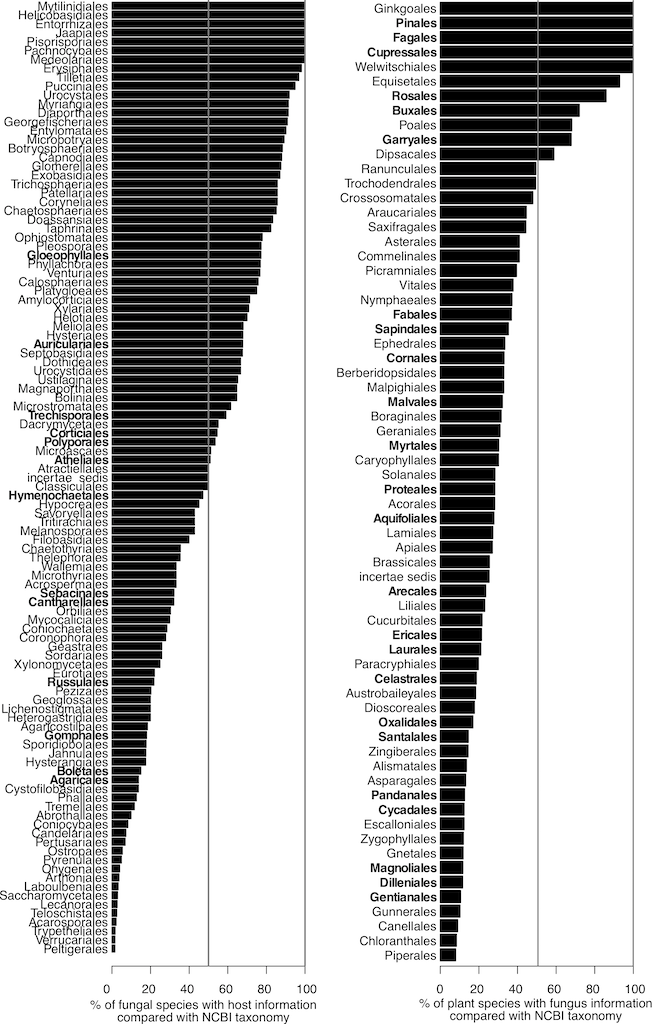


**Fig. S8** Overview of query results using R package “rusda” based on the Fungus-Host Distribution Database (FHDD) using 29,591 Dikarya and 105,350 Spermatophyta species as input. A) Number of fungal species with plant (host) information (one or more records). B) Number of plant species with fungal information. Fungal orders used in this study in bold. Woody plant orders in bold, based on FitzJohn *et al.* [10].

Table S1 Best partition scheme found by IQ-Tree [11,12].

DNA, 5_8s_28s = 1-7098, 7099-14016

DNA, 18s = 14017-18413

DNA, rpb1 = 18414-25159

DNA, rpb2 = 25160-29754

DNA, atp6 = 29755-33001

DNA, tef1 = 33002-37466

Table S2 Tip state frequencies of white and brown rot specialization based on different thresholds of host association [%]. Exclusivity: 100% = gymnosperm specialist, 0% = angiosperm specialist. 90-10 specialization: > 90% = gymnosperm specialist, < 10% = angiosperm specialist. A = Angiosperm; G = Gymnosperm; Gen = Generalist; W = White rot; B = Brown rot.

| Specialisation threshold | A-B | A-W | G-B | G-W | Gen-B | Gen-W |
| --- | --- | --- | --- | --- | --- | --- |
| 90-10 Specialization | 35 | 530 | 36 | 169 | 55 | 332 |
| 100-0 Exclusivity | 31 | 428 | 26 | 145 | 69 | 458 |

Table S3 Re-classification of taxonomic orders based on Binder et al., 2005; Hibbett et al., 2014; Larsson, Larsson, & Kõljalg, 2004.

| Re-Classification | | |
| --- | --- | --- |
| NCBI Taxonomy | Order | Genus |
| Polyporales | Agaricales | Grifola |
| Atheliales | Agaricales | Plicaturopsis |
| Polyporales | Agaricales | Xerotus |
| Polyporales | Amylocorticiales | Anomoloma |
| Polyporales | Amylocorticiales | Anomoporia |
| Russulales | Atheliales | Cristinia |
| Amylocorticiales | Atheliales | Irpicodon |
| Atheliales | Corticiales | Athelopsis |
| Hymenochaetales | Corticiales | Basidioradulum |
| Polyporales | Corticiales | Byssomerulius |
| Polyporales | Corticiales | Candelabrochaete |
| Polyporales | Corticiales | Crustoderma |
| Agaricales | Corticiales | Cylindrobasidium |
| Russulales | Corticiales | Dendrophora |
| Polyporales | Corticiales | Dentocorticium |
| Polyporales | Corticiales | Hyphoderma |
| Hymenochaetales | Corticiales | Hyphodontia |
| Atheliales | Corticiales | Hypochniciellum |
| Polyporales | Corticiales | Hypochnicium |
| Russulales | Corticiales | Laurilia |
| Polyporales | Corticiales | Phlebia |
| Polyporales | Corticiales | Pulcherricium |
| Agaricales | Corticiales | Radulomyces |
| Polyporales | Corticiales | Rhizochaete |
| Hymenochaetales | Corticiales | Schizopora |
| Polyporales | Corticiales | Scopuloides |
| Cantharellales | Corticiales | Sistotrema |
| Hymenochaetales | Corticiales | Tubulicrinis |
| Polyporales | Gloeophyllales | Neolentinus |
| Russulales | Gomphales | Ramaricium |
| Polyporales | Hymenochaetales | Phaeolus |
| Polyporales | Hymenochaetales | Resinicium |
| Amylocorticiales | Polyporales | Ceraceomyces |
| Corticiales | Russulales | Galzinia |
| Agaricales | Russulales | Granulobasidium |
| Polyporales | Russulales | Lopharia |

Table S4 Table of reference species and association numbers from NCBI

| Subdivision | Species | 5.8S rRNA | 28S rRNA | 18S rRNA | rpb1 | rpb2 | tef1 | atp6 |
| --- | --- | --- | --- | --- | --- | --- | --- | --- |
| Agaricomycotina | *Clavaria zollingeri* |  |  | AY657008 |  |  |  |  |
| Agaricomycotina | *Coprinus comatus* | AF438568 | AY635772 | AY665772 | AY857983 | AY780934 | AY881026 | DQ131603 |
| Agaricomycotina | *Clmacodon sepentrionalis* |  |  | AY705964 |  |  |  |  |
| Agaricomycotina | *Ramaria rubella* |  |  | AY707095 |  |  |  |  |
| Agaricomycotina | *Calocera cornea* |  |  | AY771610 |  |  |  |  |
| Agaricomycotina | *Rhizoctonia solani* |  |  | Genome |  |  |  |  |

**Table S5** Phylogenetic and normal linear regression on the number of angio- and gymnosperm hosts between white and brown rot species. Note that the number of fungal species was log_10_-transformed. Significant effects were highlighted in bold. We present only results, which were based on the lambda model as a model of covariance among species. We compared all available models in the function *phylolm* from the R package “phytools” [16]. The lambda model was the best model based on AIC scores (not shown).

|  |  | **phylogenetic linear model** | | | | **linear model** | |
| --- | --- | --- | --- | --- | --- | --- | --- |
|  |  | t value | p value | lambda | t value 100 trees | t value | p value |
| Angiosperm | Intercept | **2.93** | **0.003** | 0.36 | **3.73 (3.70 - 3.76)** | **12.74** | **0.000** |
|  | decay mode - WR vs. BR | 1.23 | 0.221 |  | 1.32 (1.30 - 1.35) | 1.84 | 0.066 |
| Gymnosperm | (Intercept) | **2.81** | **0.005** | 0.62 | **3.88 (3.84 - 3.93)** | **17.85** | **0.000** |
|  | decay mode - WR vs. BR | –1.60 | 0.111 |  | –1.66 (-1.71 - –1.62) | **–7.86** | **0.000** |

**Table S6** The fit of three alternative models of host association evolution in white and brown rot lineages of Agaricomycetes based on the **exclusivity** coding (100-0). The best model (shown in bold), based on Akaike weights (*w)*, allowed only intermediate host transitions (paths), no brown rot to white rot reversals (no BR reversals) and assumed equal probabilities for the root state among the six tip states.

| **Model** | -Ln L | AIC | Δ AIC | *w* |
| --- | --- | --- | --- | --- |
| Uncorrelated, ER | –1870,83 | 3743,65 | 1354,33 | 0,000 |
| Uncorrelated, ARD | –1180,64 | 2421,27 | 31,95 | 0,000 |
| Correlated hosts, ER | –1774,09 | 3550,19 | 1160,86 | 0,000 |
| Correlated hosts, ARD | –1183,56 | 2395,12 | 5,80 | 0,046 |
| Correlated hosts – norev, ER | –1941,71 | 3885,41 | 1496,09 | 0,000 |
| **Correlated hosts - norev, ARD, root = equal** | –1183,66 | 2389,32 | 0,00 | 0,830 |
| Correlated hosts - norev, ARD, root = white rot | –1185,56 | 2393,12 | 3,79 | 0,124 |

**References**

1. Smith SA, Donoghue MJ. Rates of molecular evolution are linked to life history in flowering plants. Science. 2008;322:86–9.

2. Smith SA, Beaulieu JM, Donoghue MJ. Mega-phylogeny approach for comparative biology: an alternative to supertree and supermatrix approaches. BMC Evol. Biol. 2009;9:37.

3. Zanne AE, Tank DC, Cornwell WK, Eastman JM, Smith SA, FitzJohn RG, et al. Three keys to the radiation of angiosperms into freezing environments. Nature [Internet]. 2013;506:89–92. Available from: http://www.nature.com/doifinder/10.1038/nature12872

4. Farr DF, Rossman AY, Palm ME, McCray EB. Fungal databases, systematic mycology and microbiology laboratory [Internet]. ARS, USDA. 2012. Available from: http://nt.ars-grin.gov/fungaldatabases/

5. Heibl C. The megapera package: Large phylogenetic dataset assembly in R. Modern Phylogenetic Comparative Methods and their application in evolutionary biology. Seville, Spain,. 11-15.11.2014. 2014.

6. Kohler A, Kuo A, Nagy LG, Morin E, Barry KW, Buscot F, et al. Convergent losses of decay mechanisms and rapid turnover of symbiosis genes in mycorrhizal mutualists. Nat. Genet. 2015;47:410–5.

7. Nagy LG, Riley R, Tritt A, Adam C, Daum C, Floudas D, et al. Comparative Genomics of Early-Diverging Mushroom-Forming Fungi Provides Insights into the Origins of Lignocellulose Decay Capabilities. Mol. Biol. Evol. 2015;msv337.

8. Floudas D, Binder M, Riley R, Barry K, Blanchette RA, Henrissat B, et al. The Paleozoic Origin of Enzymatic Lignin Decomposition Reconstructed from 31 Fungal Genomes. Science (80-. ). 2012;336:1715–9.

9. Harrell Jr FE, with contributions from Charles Dupont, many others. Hmisc: Harrell Miscellaneous [Internet]. 2017. Available from: https://cran.r-project.org/package=Hmisc

10. FitzJohn RG, Pennell MW, Zanne AE, Stevens PF, Tank DC, Cornwell WK. How much of the world is woody? J. Ecol. Wiley Online Library; 2014;

11. Chernomor O, Von Haeseler A, Minh BQ. Terrace Aware Data Structure for Phylogenomic Inference from Supermatrices. Syst. Biol. 2016;65:997–1008.

12. Nguyen LT, Schmidt HA, Von Haeseler A, Minh BQ. IQ-TREE: A fast and effective stochastic algorithm for estimating maximum-likelihood phylogenies. Mol. Biol. Evol. 2015;32:268–74.

13. Larsson K-H, Larsson E, Kõljalg U. High phylogenetic diversity among corticioid homobasidiomycetes. Mycol. Res. Cambridge Univ Press; 2004;108:983–1002.

14. Binder M, Hibbett DS, Larsson K-H, Larsson E, Langer E, Langer G. The phylogenetic distribution of resupinate forms across the major clades of mushroom-forming fungi (Homobasidiomycetes). Syst. Biodivers. Cambridge Univ Press; 2005;3:113–57.

15. Hibbett DS, Bauer R, Binder M, Giachini AJ, Hosaka K, Justo A, et al. Agaricomycetes. In: Esser K, editor. Mycota A Compr. Treatise Fungi as Exp. Syst. Basic Appl. Res. Springer; 2014. p. 373–429.

16. Tung Ho LS, Ané C. A linear-time algorithm for gaussian and non-gaussian trait evolution models. Syst. Biol. 2014;63:397–408.
